# Magneto-Optics of Anisotropic Exciton Polaritons in Two-Dimensional Perovskites

**DOI:** 10.1021/acs.nanolett.5c00910

**Published:** 2025-05-13

**Authors:** Jonas K. König, Jamie M. Fitzgerald, Ermin Malic

**Affiliations:** Fachbereich Physik, 9377 Philipps-Universität, Marburg 35032, Germany

**Keywords:** exciton polaritons, dark excitons, anisotropic polaritons, magneto polaritons, 2D perovskites

## Abstract

Layered two-dimensional (2D) organic–inorganic perovskite semiconductors support strongly confined excitons that offer significant potential for ultrathin polaritonic devices due to their tunability and huge oscillator strength. The application of a magnetic field has proven to be an invaluable tool for investigating the exciton fine structure observed in these materials, yet the combination of an in-plane magnetic field and the strong coupling regime has remained largely unexplored. In this work, we combine microscopic theory with a rigorous solution of Maxwell’s equations to model the magneto-optics of exciton polaritons in 2D perovskites. We predict that the brightened dark exciton state can enter the strong coupling regime. Furthermore, the magnetic-field-induced mixing of polarization selection rules and the breaking of in-plane symmetry lead to highly anisotropic polariton branches. This study contributes to a better understanding of the exciton fine structure in 2D perovskites and demonstrates the cavity control of anisotropic and polarization-sensitive exciton polaritons.

## Graphical abstract

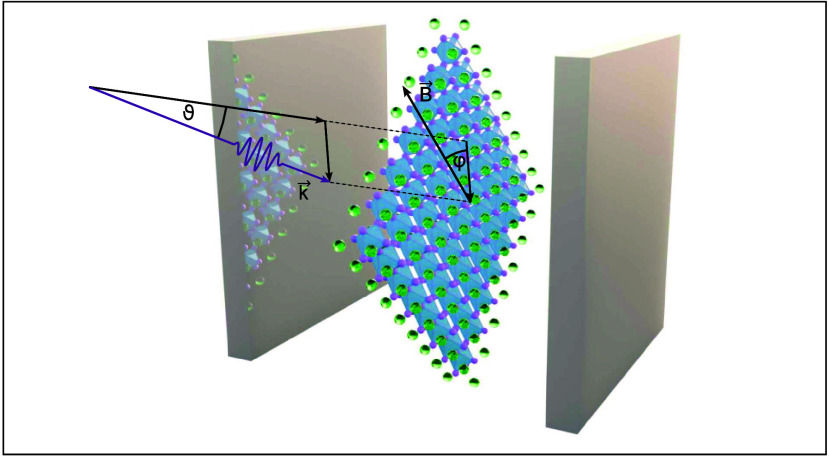

Layered two-dimensional (2D) hybrid organic–inorganic metal halide perovskites have attracted a great deal of interest in recent years due to their improved environmental stability and superior tunability compared to conventional three-dimensional counterparts.^1,2^ They offer potential applications in ultrathin light emitters,^3,4^ photovoltaics,^5,6^ photodetectors,^7^ and chiral optoelectronics.^8^ Composed of an inorganic perovskite layer sandwiched between two layers of organic spacers acting as potential barriers, they form intrinsic 2D quantum well heterostructures.^9^ These naturally stack on top of one another to form a single-crystal slab, significantly enhancing the light–matter interaction through collective effects without modifying electronic properties,^10^ in contrast to transition metal dichalcogenides.^11^ Their remarkable optical properties are governed by tightly bound excitons confined to the plane of the inorganic layer^12^ and show a rich exciton fine structure that comprises bright triplet and dark singlet states.^13,14^ Application of a twist angle^15^ and modification of the organic spacer^16^ have recently been shown to provide a tunable exciton oscillator strength and spacing, respectively. Emission studies of both nanocrystal and 2D lead iodide perovskites have revealed deviations of the exciton distribution from Boltzmann statistics, resulting in surprisingly intense emission from higher-energy bright states even at cryogenic temperatures.^17–19^ This is a direct consequence of an exciton relaxation bottleneck caused by a mismatch between the dark–bright exciton splitting and the energy of the involved optical phonons.^20^ There has been controversy in the literature regarding the energetic ordering of the dark and lowest bright states.^21,22^ The application of magneto-optical spectroscopy has played a key role in this debate, as it enables the direct observation of the dark state through the mixing of the singlet with a neighboring bright triplet state.^18–20,23,24^

The large oscillator strength of their bright excitons, resulting from quantum and dielectric confinement, makes 2D metal halide perovskites exceptional candidates for room-temperature exciton polaritonics.^25^ The strong coupling regime, with Rabi splittings in the range of hundreds of millielectronvolts, has been achieved for perovskites integrated as active layers with planar microcavities,^26–29^ plasmonic structures,^30,31^ cavity-free self-hybridized slabs^28,32,33^ , and photonic crystals/metasurfaces.^34–36^ Interesting cavity physics has already been explored, including polariton bottlenecks,^29^ topological polaritons,^37,38^ the optical spin Hall effect,^39^ polariton lasing^34^ and condensation.^35,40^ In addition, combining polaritonics with the application of a magnetic field has been used to tune the Berry curvature of exciton polariton bands^41^ and, in general, provides interesting opportunities for anisotropic polaritonics^42^ via the breaking of in-plane symmetry. The brightening of the dark fine structure exciton^19^ makes it relevant for photonics, offering the potential for cavity control of spin,^43^ polarization,^24,44^ and directional transport,^36,45^ which are crucial ingredients for many quantum optoelectronic applications.^23^ The photonic hybridization of different exciton states, both with and without an applied magnetic field, presents an exciting strategy to control, characterize, and visualize the still-debated exciton fine structure in 2D perovskites.

Based on a microscopic, material-specific, and predictive many-particle theory, we investigate the exciton fine structure of exemplary layered (PEA)_2_PbI_4_ perovskites,^46^ consisting of a single lead iodide perovskite layer sandwiched between two layers of phenylethylammonium as organic spacers. We combine the Wannier equation, which provides microscopic access to exciton characteristics, with a full-wave solution of Maxwell’s equations to describe exciton polaritons in 2D perovskites integrated within a Fabry–Pérot microcavity. Employing an in-plane magnetic field gives rise to rich optical selection rules, brightening the dark state^19^ and even potentially enabling it to enter the strong coupling regime. We study the effects of the magnetic field in terms of Rabi splitting and absorption of the polariton landscape. In particular, we observe interesting superimposed anisotropic polariton branches due to the magnetic field breaking the in-plane symmetry. Our work provides a first prediction of the magnetoabsorption of exciton polaritons in 2D perovskites formed from the brightened dark state, highlighting their experimental signatures and tunability using the magnetic-field strength and cavity length. This is relevant for potential ultrathin and tunable polarization-sensitive photonic devices as well as for the characterization and visualization of the exciton fine structure in 2D perovskites.

To obtain the exciton energy landscape, we first solve the Wannier equation,^20^ which provides microscopic access to excitonic wave functions and binding energies. The short- and long-range exchange interaction between the electrons and holes is then converted into the exciton picture, and the resulting Hamiltonian is diagonalized (see the section S.1.2 in the Supporting Information for further details). This results in an exciton fine structure energy landscape, including rich optical selection rules.^20^ With this approach, we are able to accurately describe previously observed features of the exciton fine structure of a 2D (PEA)_2_PbI_4_ perovskite layer.^19,24,47^ To model the optics of the perovskite layer, both in vacuum and within a Fabry–Pérot microcavity, we solve Maxwell’s equations using the scattering matrix (S-matrix) method, which is suitable for layered media that are spatially homogeneous in the plane^48^ (see section S.1.3). Excitons and their selection rules are included via a dispersive and anisotropic dielectric tensor

$$\varepsilon \left(\omega \right)=\left(\begin{array}{ccc} \varepsilon_{B}+\chi_{x}\left(\omega \right) & 0 & 0\\ 0 & \varepsilon_{B}+\chi_{y}\left(\omega \right) & 0\\ 0 & 0 & \varepsilon_{B} \end{array}\right)$$ 1

where *ε*_B_ is the dielectric background of the perovskite and χ_*x*_ and χ_*y*_ are the frequency-dependent response functions of the material in the in-plane *x* and *y* directions, respectively. The latter is given by^49^

$$\chi_{x\left(y\right)}\propto \underset{\mu }{\sum }\frac{\hbar \gamma_{\mu }^{x\left(y\right)}\left(\vartheta =0^{{}^{\circ}}\right)}{\left(E_{\mu }-\hbar \omega \right)-i\hbar \Gamma_{\mu }}$$ 2

where *E*_μ_ is the energy of the μth exciton, *ℏ*Γ_μ_ is the corresponding exciton scattering rate, and *ℏγ*_μ_^*x*(*y*)^ is the exciton radiative decay in the *x*(*y*) direction, which determines the oscillator strength (Figure S1). Changing the momentum components of the incoming photon allows us to vary both the angle of incidence, ϑ, and the azimuth angle, φ, with respect to the magnetic field, as shown in Figure 1a. For the sake of simplicity, we focus only on TE-polarized light, i.e., where the polarization of the light is purely in the *x–y* plane, and the excitonic contribution from the out-of-plane *z* component can be ignored.

**Figure 1:**
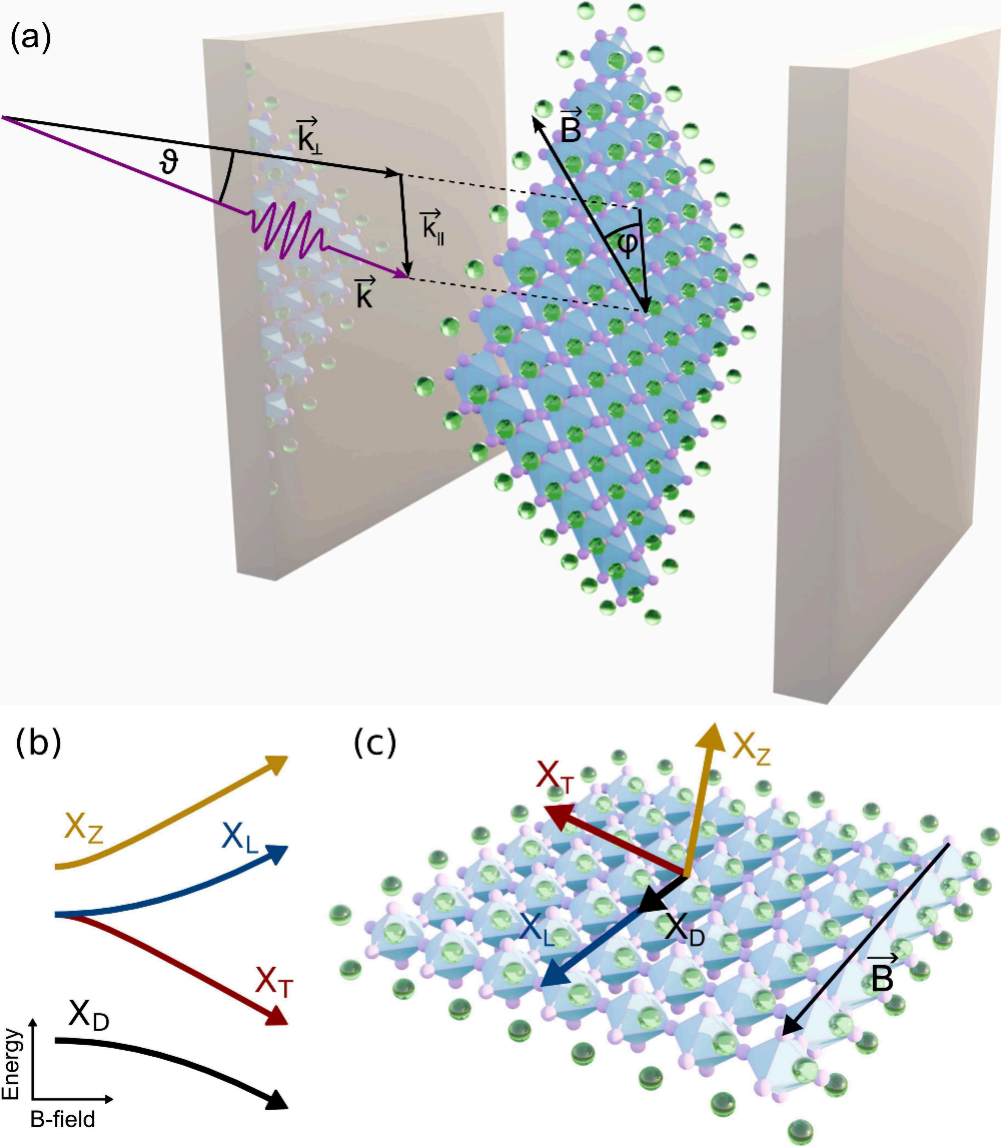
(a) Schematic figure of a 2D PEA_2_PbI_4_ perovskite slab integrated within a Fabry–Pérot cavity. The photon (purple arrow) is incident at an angle ϑ and momentum **k**, where **k**_⊥_ (**k**_∥_) denotes the perpendicular (parallel) component with respect to the perovskite layer. There is an in-plane magnetic field **B** with an azimuth angle φ relative to **k**_∥_. (b) Magnetic-field dependence of the four exciton fine structure states: dark (X_D_), the two bright (X_T_ and X_L_), and gray exciton (X_Z_). (c) Direction of the corresponding transition dipole moment for the four states in the presence of a magnetic field. While X_D_ and X_L_ are longitudinally polarized, X_T_ and X_Z_ are transversally polarized with respect to the magnetic field. The arrow length denotes the respective magnitudes of the oscillator strength.

We consider a microcavity consisting of a pair of Bragg mirrors with a single perovskite quantum well in the center (Figure 1). Using the S-matrix method, with microscopic input from the Wannier equation, we calculate the linear optical spectra of the combined system. While this provides exact solutions to Maxwell’s equations, it does not give access to the Hopfield coefficients, which are necessary for a detailed understanding of the constituent nature and decay channels of each polariton.^50,51^ We therefore extract the exciton polariton energies from the calculated reflection, and then fit the resulting dispersion manifolds to a two-exciton, one-photon Hopfield model.^50,52^ This provides microscopic access to the cavity photon–exciton coupling strength, *g*_μ_, and the Hopfield coefficients, *U*_μ_^*n*^. The Hopfield matrix is given by

$$\begin{array}{cc} H & =\left(\begin{array}{ccc} E^{\left(C\right)} & g_{1} & g_{2}\\ g_{1} & E_{1}^{\left(X\right)} & 0\\ g_{2} & 0 & E_{2}^{\left(X\right)} \end{array}\right) \end{array}$$ 3

where *E*_μ_^(X)^ is the energy of the μth exciton and *E* ^(C)^ denotes the energy of the bare cavity photon, which is extracted from an S-matrix simulation ignoring excitonic effects.

To further analyze the results, we use coupled mode theory, which is appropriate for describing the coupling of low-loss material resonances and high-*Q* photonic modes to each other and to external ports^53^ (see section S.1.4). The absorption of a single exciton μ and polarization σ is given by the Elliott formula^49^

$$A_{\mu }^{\sigma }\left(\omega \right)=\frac{2\hbar \Gamma_{\mu }\hbar \gamma_{\mu }^{\sigma }}{{\left(E_{\mu }^{\left(X\right)}-\hbar \omega \right)}^{2}+{\left(\hbar \Gamma_{\mu }+\hbar \gamma_{\mu }^{\sigma }\right)}^{2}}$$ 4

The polaritonic Elliott formula has the exact same form^50^ but instead uses the respective polariton decay rates (see eq S.5c). For a symmetric system excited from one port, the absorption has an upper bound^54^ of 0.5, which is reached under the critical coupling condition^51,55^ of γ = Γ, as one can see from eq 4.

We use the approach presented above to investigate the exciton fine structure and absorption in 2D perovskites under an applied in-plane magnetic field both with and without integration into a Fabry–Pérot cavity. Solving the Wannier equation, we obtain a binding energy of approximately 220 meV for the 4-fold degenerate exciton, originating from the possible spin configurations of electrons and holes,^14^ in excellent agreement with prior theoretical^20,56^ and experimental^46,57^ studies. Including the exchange interaction, we find that the four degenerate spin states split in energy. The energetically lowest singlet state is optically dark^18,19^ and is therefore labeled the dark state, X_D_. Furthermore, there are two degenerate circularly polarized bright states, X_T/L_, and an out-of-plane polarized gray state, X_Z_.^10,47^ If an in-plane magnetic field is applied (Voigt configuration), these states further mix, modifying their energy and optical selection rules.^19^ Their energy shift as a function of the magnetic-field strength is sketched in Figure 1b. Level repulsion causes the dark state to shift down in energy, the gray exciton to shift up, and the two degenerate bright states to split apart. The previously circularly polarized states become linearly polarized,^24^ where X_T_ is orthogonal (transversal) to the magnetic field but no longer fully in-plane, while X_L_ is parallel (longitudinal). Furthermore, the dark state brightens and becomes polarized along the magnetic-field direction due to field-induced mixing with the X_L_ state as a consequence of the spin selection rules.^58^ Finally, the gray state is also transversally polarized, but it is no longer strictly oriented out-of-plane due to mixing with the X_T_ state, allowing it to be optically accessed with TE-polarized light. The orientation of the transition dipole moments with respect to the *B* field is illustrated in Figure 1c.

According to these selection rules, when the bare perovskite slab is excited with TE-polarized light in the absence of a magnetic field, the response is independent of azimuth angle φ and only a single absorption peak at the energy of the degenerate bright states is observed. In contrast, in the presence of a high magnetic field, the optical response is crucially dependent on φ. When φ = 0° (i.e., when the electric field of the light is fully in-plane and orthogonal to the applied magnetic field), only the transversal states, X_T_ and X_Z_, can couple to the light via their in-plane component.^19^ Considering a magnetic-field strength of 50 T, this leads to two absorption peaks at small angles of incidence, as illustrated in Figure 2a. For larger angles of ϑ, the two peaks merge, and there is a strong absorption approximately at the X_L_ energy. A closer analysis of the two peaks reveals that they broaden and overlap, creating the impression of a single peak between them that coincides with X_L_, as illustrated in Figure 2b. For TE-polarized light, radiative decay *ℏγ*_μ_ of a given exciton state μ can be shown to scale with^59^ 1/cos­(ϑ) and thus diverges as ϑ limits toward 90°.^60^ Consequently, the full width at half-maximum of the peak, given by 2­(*ℏ*Γ_μ_ + *ℏγ*_μ_), also increases rapidly as ϑ approaches grazing angles. Surprisingly, as the radiative decay of the exciton diverges, the absorption decreases to zero, as shown in Figure 2c. This occurs because the difference between radiative and material-based decay rates increases with ϑ (Figure 2d), moving the system away from the critical coupling regime and, therefore, decreasing the absorption.

**Figure 2:**
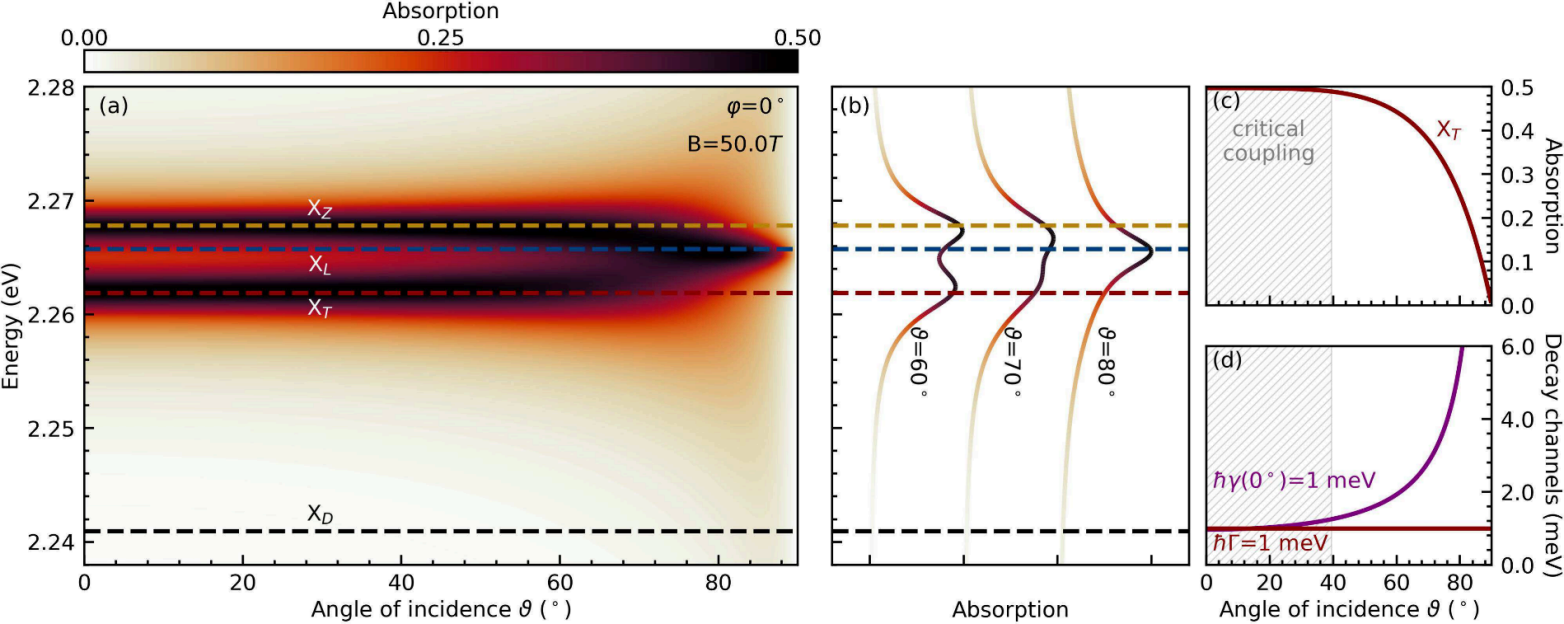
(a) Absorption of a 2D PEA_2_PbI_4_ perovskite layer as a function of the photon energy and angle of incidence for *B* = 50 T and φ = 0°. The horizontal dashed lines indicate the energies of the four exciton fine structure states. As we consider TE-polarized light, i.e., a polarization perpendicular to the magnetic field at φ = 0°, only X_T_ and X_Z_ excitons can couple to light at this orientation, as shown in Figure 1c. (b) Line cuts of the absorption spectrum at specific larger angles of incidence. (c) Absorption along the energy of X_T_. (d) Radiative (*ℏγ*) and material-based (*ℏ*Γ) decay channels for X_T_. The shaded area indicates where the two decay channels have a relative difference of less than 5%, marking the critical coupling region with maximal absorption.

The integration of a perovskite slab into a microcavity, combined with the brightening of the dark state in the presence of an in-plane magnetic field, presents a potential strategy to investigate the still-debated energetic ordering of the exciton fine structure states in these materials. Furthermore, the anisotropy induced by the magnetic field is expected to have a dramatic impact on polariton optics. In particular, by variation of azimuth angle φ, the projection of the electric field onto the transition dipole moment of the various fine structure states can be altered relative to the applied in-plane magnetic-field orientation, leading to a rich anisotropic polariton dispersion that can be selectively probed with the incident beam angle. The polariton landscape in the absence of the magnetic field is shown in Figure S.5. Here, we focus on the case of magneto-optics. Starting with φ = 0°, where the electric field is oriented perpendicular to the applied magnetic field, only the two excitons, X_T_ and X_Z_, can couple to the incident laser, similar to the bare perovskite case. This results in three polariton branches, lower (LP), middle (MP), and upper polariton branches (UP) (cf. Figure 3a). As the middle branch is sandwiched between almost energetically degenerate states X_T_ and X_Z_, it is nearly flat and therefore barely visible in absorption.^50^ As a result, only one large apparent Rabi splitting is observed instead of the two splittings typically observed for a two-exciton, one-photon system. However, the absorption of the middle branch increases with even greater magnetic fields as the separation between X_T_ and X_Z_ increases.

**Figure 3:**
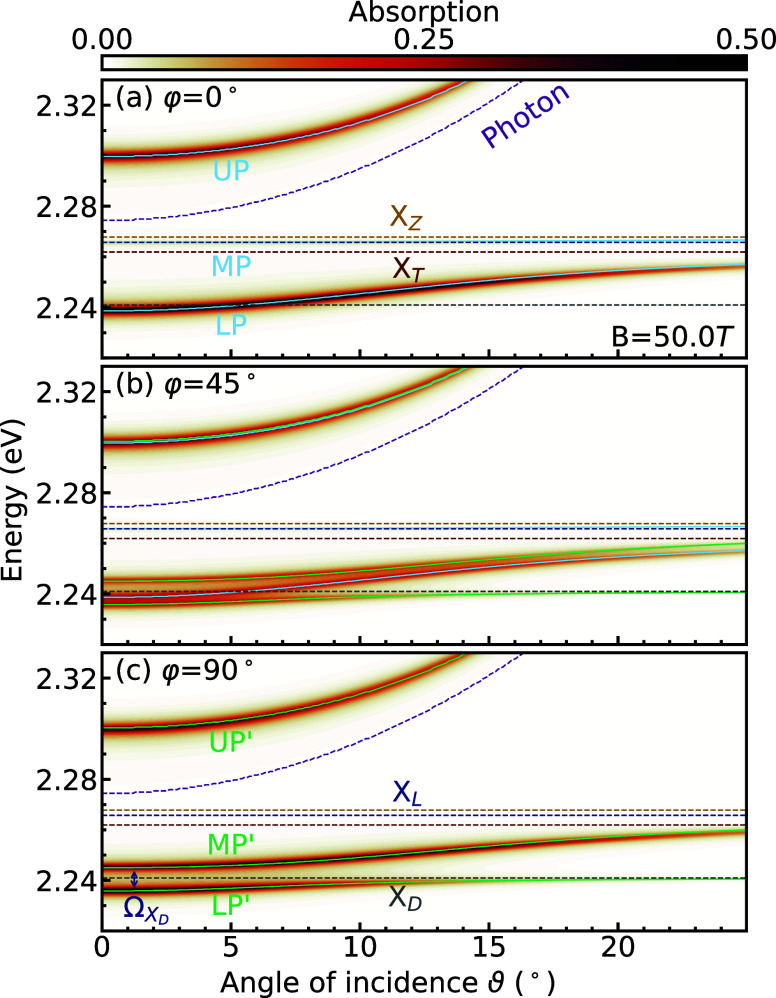
(a–c) Absorption of a 2D (PEA)_2_PbI_4_ perovskite layer integrated within a Fabry–Pérot microcavity as a function of the photon energy and angle of incidence for *B* = 50 T and different azimuth angles φ. The horizontal dashed lines indicate the exciton energies. Depending on φ, different polariton branches (denoted by LP, MP, and UP for φ = 0° and LP′, MP′, and UP′ for φ = 90°) appear as a result of the selection rules of the respective excitonic states. The small vertical arrow in panel c denotes the Rabi splitting of the dark state, Ω_X_D__, which brightens in the presence of a magnetic field.

When φ = 90°, where the electric field is oriented parallel to the applied magnetic field, only X_L_ and X_D_ can couple to the light. This leads again to three polariton branches (LP′, MP′, and UP′) (Figure 3c). As X_L_ and X_D_ are well separated in energy, the middle polariton is no longer flat, and we observe two Rabi splittings: one around X_L_ and a smaller splitting around X_D_. The latter is labeled as Ω_X_D__ and defined as the difference between LP′ and MP′ at ϑ = 0°. The upper polariton branch (UP) is approximately independent of φ and hence very similar for the 0° and 90° cases. This is because the combined oscillator strength of X_Z_ and X_T_ is approximately equal to that of X_L_, due to the conservation of oscillator strength in the presence of Zeeman splitting.^20^

Applying a fit using the Hopfield model (eq 3) gives a Rabi splitting of Ω_X_D__ = 9 meV for the dark state at *B* = 50 T, while for X_L_ the splitting is 54 meV. In comparison, the Rabi splitting of the X_T_ and X_Z_ states in the φ = 0° case is 60 meV. The detuning was chosen such that the LP in Figure 3a coincides approximately with the dark state, leading to a Rabi splitting around the dark state, Ω_X_D__, to be at ϑ = 0°, as indicated by the small vertical arrow in Figure 3c. Smaller cavity lengths would increase the energy of the cavity photon mode, thereby detuning the cavity from the X_D_ energy and leading to a smaller Rabi splitting. In contrast, a longer cavity length would decrease the cavity photon energy, shifting the Rabi splitting to larger angles of incidence. These results constitute a first prediction that at certain incident laser orientations, the brightened low-energy dark state can enter the strong coupling regime and be observed by magneto-optical measurements. This paves the way toward cavity control of the exciton fine structure in 2D perovskites.

An alternative approach to brightening a dark material, effective even at zero magnetic field, is to exploit the large Rabi splitting to shift the lower polariton (composed of the two degenerate bright states) energetically below the dark exciton, thereby enhancing the photoluminescence intensity. Such an approach has been demonstrated for tungsten-based TMD monolayers.^61^ In Figure S.7, we show that integrating a perovskite slab into a cavity results in the lower polariton becoming the energetically lowest state, leading to a much higher emission yield compared to that of a bare perovskite slab.

Interestingly, at intermediate angle φ = 45°, we do not observe a smaller Rabi splitting around the brightened dark state (Figure 3b), as might be naively expected from considering only the projection of the oscillator strength. Instead, we observe a superposition of the two previously discussed limiting cases of φ = 0° and φ = 90° with unchanged Rabi splittings but altered absorption. The upper branch still has the same absorption due to the near degeneracy of UP and UP′, while the other branches have their absorption approximately halved. This behavior of superimposed Rabi splittings is related to a magnetic-field-induced anisotropy of the dielectric tensor, which arises from the two inequivalent dipoles pointing in different directions. Similar results have previously been observed in oriented molecular aggregates^62^ and 2D naturally anisotropic TMD (ReS_2_) layers.^63^ In the Hopfield model, this means that there are two degenerate cavity modes polarized orthogonal to each other, which oscillate perpendicular (parallel) to the magnetic field and couple only to X_T_ and X_Z_ (only to X_D_ and X_L_). Therefore, this six-branch system decouples into two three-branch subsystems, oriented perpendicular and parallel to the magnetic field, respectively, and exhibiting the same cavity–exciton couplings as before. The observed magnetic field and cavity control of the polariton landscape, particularly the modification of the Rabi splitting relative to typical phonon energies, presents opportunities to manipulate optics^56,64^ and relaxation in these materials,^20,65^ with relevance, e.g., to polariton lasing.^66^ The anisotropic polariton bands will lead to a direction-dependent relaxation, with the middle polariton branch anticipated to play an important role in dictating relaxation from higher-energy states to the lower polariton branch.^67^

Now, we explore the tunability of the system with respect to the applied in-plane magnetic field. With an increase in field strength, the oscillator strength is transferred from X_L_ to X_D_ (see section S.1.2). Therefore, Rabi splitting Ω_X_D__ around the dark state is expected to also grow. We find that Ω_X_D__ scales linearly with the magnetic field (Figure 4a). The oscillator strength of the dark state scales quadratically with the magnetic field, provided that Zeeman splitting μ_B_ *B* is small compared to the energy difference between the X_L_ and X_D_ states.^58^ Furthermore, the coupling between the exciton and cavity photon modes is proportional to the square root of the oscillator strength,^50^ explaining the observed linear behavior. The absorption as a function of the magnetic-field strength is provided in Figure S.6.

**Figure 4:**
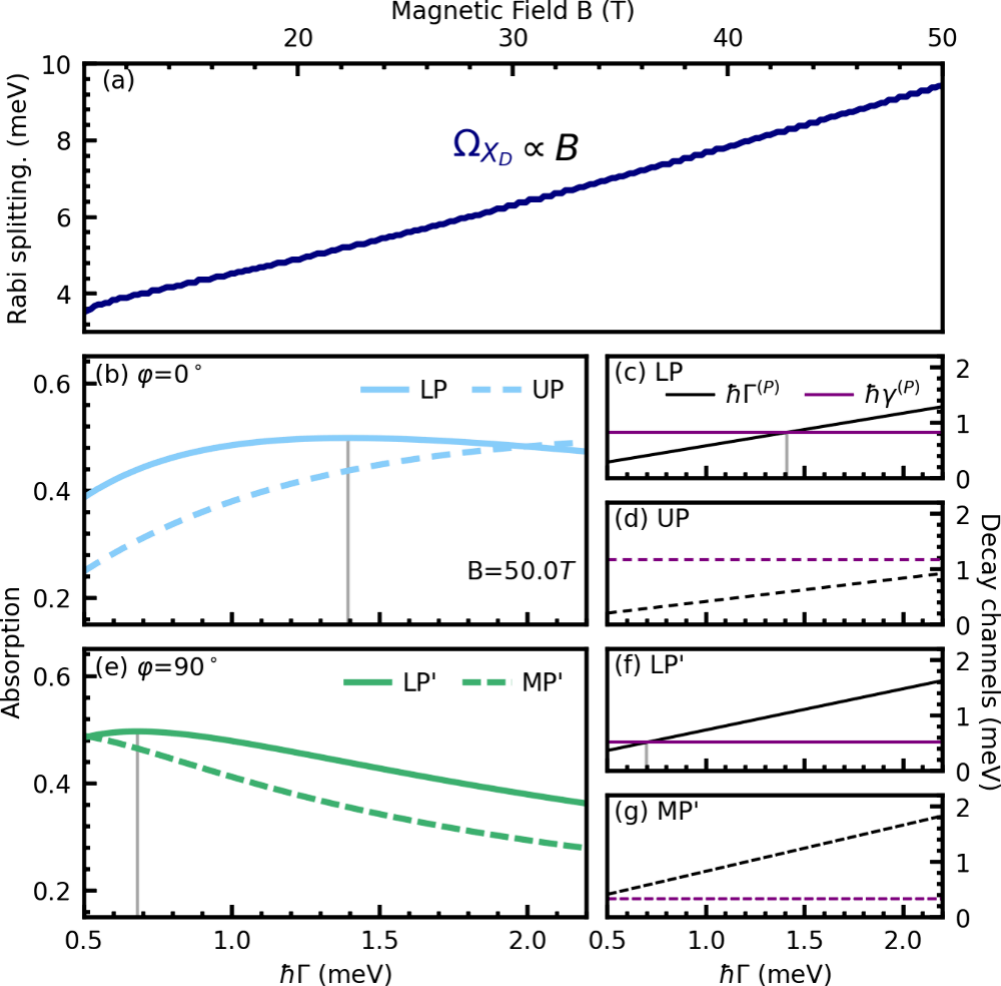
(a) Rabi splitting, Ω_X_D__, stemming from the avoided crossing between the dark state and the photon energy, as a function of magnetic-field strength *B* (see the arrow in Figure 3c). (b) Resonant absorption of the lower (LP) and upper (UP) polariton branches from Figure 3a (φ = 0°) at ϑ = 0° as a function of excitonic line width *ℏ*Γ. The vertical gray line indicates the point at which the absorption reaches a peak value of 0.5. (c and d) Photonic, *ℏγ* ^(*P*)^, and excitonic, *ℏ*Γ^(*P*)^, decay channels of the LP and UP polariton branches, respectively. The vertical gray line shows where the two are equal; i.e., the critical coupling condition is met. It coincides exactly with the vertical gray line in panel b. (e) Same as panel b, but with φ = 90°, illustrating the lower (LP′) and middle (MP′) polariton branches from Figure 3c. (f and g) Photonic and excitonic decay channels of the LP′ and MP′ polariton branches, respectively.

Another way to tune the system is to change exciton line width *ℏ*Γ via, e.g., varying the temperature.^56^ The material-based loss of a polariton branch *n* is given by the sum of the line widths of all its excitonic components, weighted by the respective excitonic Hopfield coefficients *ℏ*Γ_*n*_^(*P*)^ = ∑_μ_ *ℏ*Γ|*U*_μ_^*n*^|^2^.^50,51^ Similarly, the radiative decay is given by the cavity mode line width, *ℏκ*, scaled by the photonic Hopfield coefficient *ℏγ*_*n*_^(*P*)^ = *ℏκ*|*U*_0_^*n*^|^2^. The different branches in Figure 3a reach a large absorption at ϑ = 0° for different *ℏ*Γ values, as illustrated in Figure 4b. For a particular polariton branch, we find that high absorption coincides with regions where the respective excitonic and photonic loss rates are similar (Figure 4c,d), i.e., fulfilling the critical coupling condition that is contained in the polaritonic Elliott formula (eq S.5c).^50,51^ For the φ = 90° case, we find qualitatively the same results (Figure 4e–g), with the main distinction from the 0° case being the different Hopfield coefficients, i.e., different light–matter compositions of the polariton branches. A plot of the Hopfield coefficients as a function of the angle of incidence can be found in Figure S.8. These results reveal how a combination of magneto-optical measurements and modeling using the Hopfield method and Elliott formula can unravel the interplay of optical selection rules and the balance of decay channels that determine the optical response of exciton polaritons in 2D perovskites.

We have revealed the rich magneto-optical response of dark and bright exciton states in 2D perovskites, both in a vacuum and when integrated within a Fabry–Pérot microcavity. In particular, we show that the dark state can enter the strong coupling regime under an applied in-plane magnetic field. Furthermore, we predict anisotropic polariton manifolds in 2D perovskites arising from a magnetic-field-induced anisotropy. We also demonstrate that the dispersion and the absorption of different polariton branches can be tuned by adjusting the azimuth angle of the light beam relative to the magnetic field. These gained insights contribute to a deeper microscopic understanding of exciton polaritons in 2D perovskites and could also be important for the technologically relevant directional tuning and polarization control of both polariton transport and emission.

## Supplementary Material



## Notes

Details on the derivation of the main equations and additional discussion of the magnetic-field-dependent polariton landscape (PDF)
