# Supplementary material for: Magneto-Optics of Anisotropic Exciton Polaritons in Two-Dimensional Perovskites
Source: Nano Lett. 2025 May 13;25(21):8519–26. doi: 10.1021/acs.nanolett.5c00910 (PMC12123678; doi:10.1021/acs.nanolett.5c00910)
Supplement: Supplementary file 1 [file nl5c00910_si_001.pdf]

Supplementary Information
Magneto-Optics of Anisotropic Exciton Polaritons in Two-Dimensional Perovskites

Jonas K. König,* Jamie M. Fitzgerald, and Ermin Malic

Fachbereich Physik, Philipps-Universität, Marburg, 35032, Germany

E-mail: jonas.koenig@physik.uni-marburg.de

S.1 Methods

S.1.1 Wannier equation

To obtain exciton energies and wavefunctions, we solve the Wannier equation[1]

$$\sum_{\mathbf{k}'} \left(\frac{\hbar^2 k'^2}{2m_r} \delta_{kk'} + V_{|\mathbf{k}-\mathbf{k}'|} \right) \Psi_n(\mathbf{k}') = E_b^n(\mathbf{k}) \Psi_n(\mathbf{k}) \;, \tag{S.1}$$

where $m_r = 0.108 m_e$ is the reduced mass of the electron and hole[2], V_q is the screened Coulomb potential, E_n are the exciton binding energies, and the corresponding wavefunctions are denoted by Ψ_n. The electron and hole masses are taken from Ref. 2 and the Keldysh approximation is used for the screened Coulomb potential[3]. The high-frequency dielectric constants of the organic spacer layer and the inorganic perovskite layer are set to[4] 3.3 and 6.1 , respectively. The thickness of the perovskite slab is set to[4] 0.636 nm.

S.1.2 Exchange interaction and optical selection rules

Considering only the 1s exciton, the spin combination of the constituent electron and hole leads to four degenerate states. These states interact with each other through the exchange interaction, as well as with an applied magnetic field via the Zeeman effect, leading to the following eigenvalue problem in the exciton basis[5]

$$\begin{pmatrix} I_Z & -g_v(B) & g_c(B) & -I_Z \\ -g_v(B) & I_r & 0 & g_c(B) \\ g_c(B) & 0 & I_r & -g_v(B) \\ -I_Z & g_c(B) & -g_v(B) & I_Z \end{pmatrix} \begin{pmatrix} D^{\uparrow\uparrow}_{\mu,\mathbf{q}} \\ D^{\uparrow\downarrow}_{\mu,\mathbf{q}} \\ D^{\downarrow\uparrow}_{\mu,\mathbf{q}} \\ D^{\downarrow\downarrow}_{\mu,\mathbf{q}} \end{pmatrix} = E^{(X)}_{\mu,\mathbf{q}}(B) \begin{pmatrix} D^{\uparrow\uparrow}_{\mu,\mathbf{q}} \\ D^{\uparrow\downarrow}_{\mu,\mathbf{q}} \\ D^{\downarrow\uparrow}_{\mu,\mathbf{q}} \\ D^{\downarrow\downarrow}_{\mu,\mathbf{q}} \end{pmatrix} , \qquad \text{(S.2)}$$

where $I_{r(Z)}$ quantify both the short- and long-range exchange interaction strength in the in-plane (out-of-plane) direction of the perovskite. Furthermore, $g_{c/v}(B) = g_{c/v}\frac{\mu_B B}{2}$, and $g_c = 2.9$ and $g_v = -1.1$ denote the g-factors in the excitonic basis[6]. At zero magnetic field, the resulting excitonic fine structure states at $|\mathbf{q}| = 0$ are given by

$$E^{(X)}_D = \Delta + E^{1s}_b , \quad E^{(X)}_T = \Delta + E^{1s}_b + I_r , \quad E^{(X)}_L = \Delta + E^{1s}_b + I_r , \quad E^{(X)}_Z = \Delta + E^{1s}_b + 2I_Z ,$$

where Δ is the bandgap energy. The respective eigenvectors are given by

$$D_D = \frac{1}{\sqrt{2}}(1,0,0,1) \ , \quad D_T = (0,1,0,0) \ , \quad D_L = (0,0,1,0) \ , \quad D_Z = \frac{1}{\sqrt{2}}(1,0,0,-1) \ .$$

Here, we fit I_r and I_Z to the experimentally obtained values for the splitting between the dark (X_D) and bright states ($X_{T/L}$), as well as between the dark and gray state (X_Z)[6].

The oscillator strength $|M^{\mu}_{\sigma}(\mathbf{B})|^2$ of each state is a linear combination of the dipole moments of each exciton, $\mathbf{d}^{cv}_{ss'}$, weighted by the respective eigenvector, and then projected

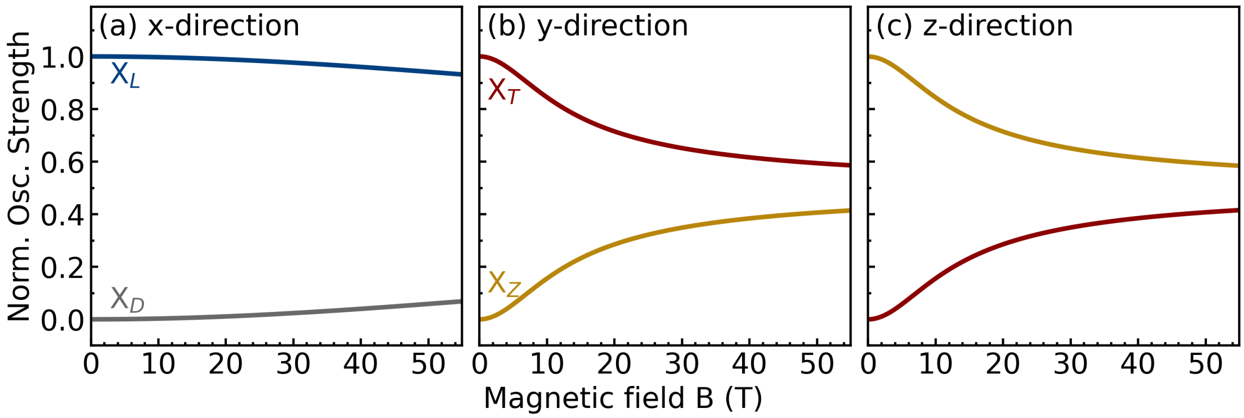

Figure S.1: (a) Oscillator strength in the x-direction of the excitonic fine structure states, X_D and X_L, which are polarized in the x-direction (longitudinal to the magnetic field). (b)-(c) Oscillator strength in the y-direction (transversal and in-plane) and the z-direction (transversal and out-of-plane) of the corresponding states, X_T and X_Z, respectively.

onto the polarization of the light[5] $\mathbf{e}_\sigma$

$$|M_\sigma^\mu|^2(\mathbf{B}) \propto |\Psi_{1s}(\mathbf{r}=0)|^2 |\mathbf{e}_\sigma \cdot \sum_{ss'} \mathbf{d}_{ss'}^{cv} D_\mu^{ss'}(\mathbf{B})| \ ,$$

where $\Psi_{1s}(\mathbf{r})$ is the wavefunction in real space, and s (s') denotes the electron (hole) spin for the respective exciton. Without a magnetic field, the dark exciton state has a zero transition dipole moment, the two degenerate bright states are circularly polarized in the xy-plane, and the gray state is polarized along the out-of-plane z direction. The magnetic field mixes these states, leading to the modified selection rules, cf. Fig. 1 of the main text. The magnetic field dependence of the oscillator strength for different polarization orientations is shown in Fig S.1. In particular, Fig. S.1(a) illustrates the transfer of oscillator strength from the bright state X_L to the dark exciton X_D with increasing magnetic field. Figures S.1(b) and (c) show the mixing of the oscillator strength for the transverse state X_T and the out-of-plane gray exciton X_Z, due to the rotation of the respective transition dipole moments around the magnetic field axis (Fig. 1 in the main text). Note that for all magnetic fields, the total oscillator strength in each polarization direction is equal to unity.

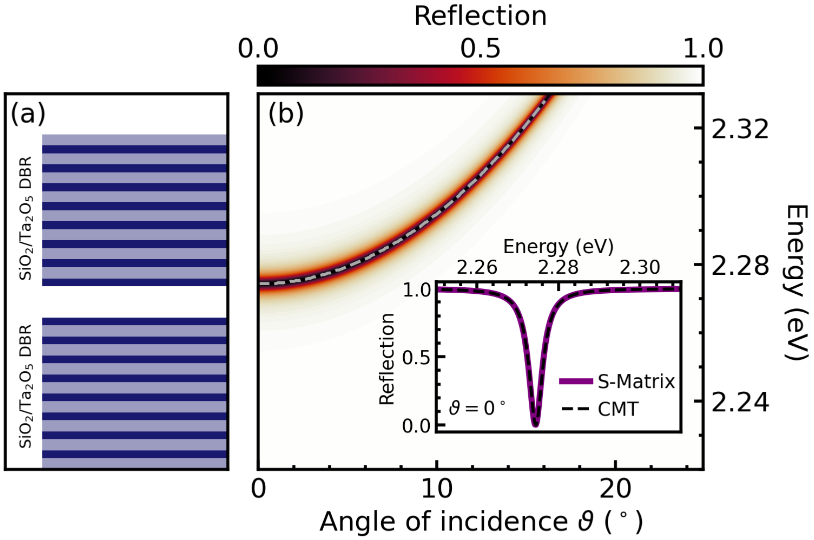

Figure S.2: (a) Schematic of the cavity used for the S-matrix simulations, constructed from two $\lambda/4$-DBR mirrors, each consisting of alternating stacked SiO_2 (light blue) and TaO_2 (dark blue) layers. (b) S-matrix simulation of the reflection of the bare Fabry-Pérot microcavity as a function of the photon energy and angle of incidence ϑ. The gray dashed line illustrates the extracted cavity photon mode energy, perfectly following the peak reflection. The inset shows a line cut at $\vartheta = 0°$ (purple solid line) and the fit using coupled mode theory (black dashed line) with the extracted photon linewidth $\hbar\kappa$.

S.1.3 Scattering matrix method

The scattering matrix (S-matrix) method is a numerical algorithm used to exactly solve Maxwell's equations for stacks of dielectric slabs with in-plane translational invariance or periodicity[7]. It provides access to the optical response of the system for any incident light angle. However, it is difficult to disentangle photonic and material contributions of a mixed light-matter state, as described by Hopfield coefficients. In particular, this is necessary to obtain access to the different decay channels associated with the constituent excitons and cavity modes. To this end, we use a Hopfield model (see Eq. 3 in the main text) using parameters extracted from S-matrix simulations.

In this work, we consider a microcavity consisting of two $\lambda/4$-DBR mirrors, each consisting of eight stacks of alternating SiO_2 and TaO_2 ($n_{SiO_2} = 1.46$ and $n_{Ta_2O_5} = 2.0770$[8]) dielectric slabs, for a total number of 16 layers per DBR. A sketch of the simulated cavity can be seen in Fig. S.2(a). The thickness of the layers was chosen such that the center

frequency of the DBR stopband aligns with the energy of the dark state. To simulate and extract the cavity mode as a function of angle of incidence, we also have to include the dielectric background of the perovskite layer in the center of the cavity, i.e., without any excitonic effects. This is because a thin dielectric slab will affect the resonance condition of the microcavity. Using a dielectric background for the perovskite layer of 1.81 [9], we obtain the reflection of the cavity, as shown in Fig. S.2(b). By fitting a Lorentzian function to the reflection (Eq. S.5a in the limit of a bare cavity), we are able to obtain the cavity linewidth $\hbar\kappa$, see the inset of Fig. S.2(b). Including the dielectric tensor given in Eqs. (1) and (2) of the main text, we can calculate the optical response of the $(PEA)_2PbI_4$ perovskite layer integrated within a microcavity, and then extract the polariton energies from the dips in reflection. These energies are then fit to the Hopfield model in Eq. (3) of the main text to obtain access to the cavity photon-exciton coupling strengths and Hopfield coefficients.

S.1.4 Coupled mode theory

Assuming high Q-factor cavity modes and small material-based losses, coupled mode theory (CMT) provides an intuitive and simple description of the coupling between different modes of the system (excitons, cavity photons) and their coupling to external ports [10]. In particular, it provides insight into calculated absorption spectra via the critical coupling condition [11–13]. Here, we detail the derivation of the polaritonic Elliott formula using classical two-port, two-resonator CMT equations. Starting from the Hamiltonian in Eq. (3) of the main text, we add an imaginary part to the exciton and cavity-photon energies on the diagonal to describe the respective loss parameters. We then obtain the following coupled dynamics of excitons and cavity photons:

$$\partial_t \begin{pmatrix} C(t) \\ X_1(t) \\ X_2(t) \end{pmatrix} = -\frac{i}{\hbar} H \cdot \begin{pmatrix} C(t) \\ X_1(t) \\ X_2(t) \end{pmatrix} = \begin{pmatrix} -i\omega^{(C)} + \kappa & -i\frac{g_1}{\hbar} & -i\frac{g_2}{\hbar} \\ -i\frac{g_1}{\hbar} & -i\omega_1^{(X)} + \Gamma & 0 \\ -i\frac{g_2}{\hbar} & 0 & -i\omega_2^{(X)} + \Gamma \end{pmatrix} \cdot \begin{pmatrix} C(t) \\ X_1(t) \\ X_2(t) \end{pmatrix}$$

Here, $X_\mu(t)$ is the mode amplitude of the μth exciton oscillating in time with the frequency $\omega_\mu^{(X)}$ and decay rate Γ. Furthermore, $C(t)$ is the amplitude of the cavity photon at the frequency $\omega^{(C)}$ with the decay rate κ. Using the Hopfield transformation[14,15] $C(t) = \sum_n P_n(t)U_0^n$ and $X_\mu(t) = \sum_n P_n(t)U_\mu^n$, we can decouple the dynamics into a set of independent differential equations. Here, $P_n(t)$ is the polariton mode amplitude of the nth branch with U_0^n and U_μ^n as the respective photonic and excitonic Hopfield coefficients.

Next, we add the coupling to external photons, i.e., the two ports corresponding to the continuum of photon states in the half-space either side of the cavity[10,15]. In a Fabry-Pérot microcavity, only the photonic component of each polariton branch can couple to the ports, i.e., excitons are not directly excited by external photons. The incoming and outgoing waves are given by

$$\left(b^+\right)^T(t) = \left(b_1^+(t), b_2^+(t)\right), \quad \left(b^-\right)^T(t) = \left(b_1^-(t), b_2^-(t)\right), \tag{S.3}$$

where $b_i^{+(-)}$ is the incoming (outgoing) wave at the ith port, as shown in Fig. S.3. The incoming waves couple to the photonic part of the polaritons through $\xi_n^T = (\xi_{n,1}, \xi_{n,2})$ for each port respectively, while the outgoing waves couple via $d_n^T = (d_{n,1}, d_{n,2})$. The direct process of the incoming waves coupling to the outgoing waves is described by a 2×2 matrix $\mathcal{C}$. As direct transmission is not possible in an idealized Fabry-Pérot cavity, we can only couple b_i^+ to b_i^- of the same port via reflection. Therefore, the matrix $\mathcal{C}$ is the negative of

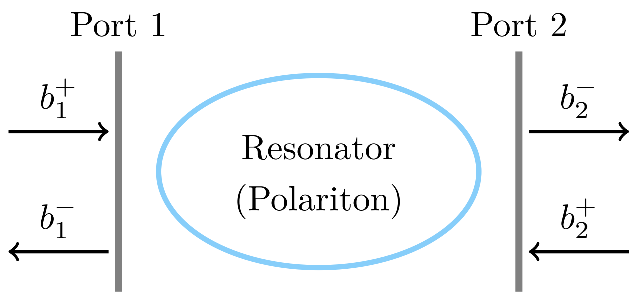

Figure S.3: Schematic of the ports 1 and 2 coupling the respective incoming waves (b_1^+, b_2^+) and outgoing waves (b_1^-, b_2^-) to the resonator, i.e., the polariton.

the identity matrix. Note that one can also add the coupling to the ports before performing the Hopfield transformation and obtain the same equations.

Exploiting time-reversal symmetry[10,16], which is valid for low absorptive losses, we obtain

$$\xi_n = d_n = \begin{pmatrix} \sqrt{2\gamma_{n,1}^{(P)}} \\ \sqrt{2\gamma_{n,2}^{(P)}} \end{pmatrix} ,$$

where $\gamma_{n,i}^{(P)}$ is the photonic decay rate of the nth polariton branch into the ith port. The sum of the polaritonic radiative decay rate into each port is equal to the total photonic-based polariton decay rate from the main text, i.e., $\gamma_{n,1}^{(P)} + \gamma_{n,2}^{(P)} = \gamma_n^{(P)}$. This results in the polariton dynamics

$$\partial_t P_n(t) = \left(-i\omega_n - \gamma_n^{(P)} - \Gamma_n^{(P)}\right) P_n(t) + \sqrt{2\gamma_{n,1}^{(P)}} b_1^+(t) + \sqrt{2\gamma_{n,2}^{(P)}} b_2^+(t) , \tag{S.4a}$$

$$\begin{pmatrix} b_1^-(t) \\ b_2^-(t) \end{pmatrix} = \begin{pmatrix} -b_1^+(t) \\ -b_2^+(t) \end{pmatrix} + \sum_n \begin{pmatrix} \sqrt{2\gamma_{n,1}^{(P)}} \\ \sqrt{2\gamma_{n,1}^{(P)}} \end{pmatrix} P_n(t) , \tag{S.4b}$$

where ω_n and $\Gamma_n^{(P)}$ are the frequency and material-based decay rates of the nth polariton branch, and $b_i^{+(-)}$ are the incoming (outgoing) fields of the ith port. The first equation describes how polaritons are excited by the incoming waves, while the second equation expresses the emission of the outgoing waves.

As we consider only excitations from one port, we set $b_2^+(t) = 0$. For well-spaced polaritons, we can ignore any overlap and therefore solve the equations independently for one polariton branch, and then simply add the final contributions together to obtain the total linear optical spectra. The Fourier transformation of Eqs. S.4a and S.4b gives the reflection

and transmission coefficients for each polariton branch:

$$r_n(\omega) = \frac{b_1^-(\omega)}{b_1^+(\omega)} = \frac{-i(\omega - \omega_n) + \left(\gamma_{n,1}^{(P)} - \gamma_{n,2}^{(P)} - \Gamma_n^{(P)}\right)}{i(\omega_n - \omega) + \gamma_n^{(P)} + \Gamma_n^{(P)}} ,$$

$$it_n(\omega) = \frac{b_2^-(\omega)}{b_1^+(\omega)} = \frac{2\sqrt{\gamma_{n,1}^{(P)}\gamma_{n,2}^{(P)}}}{i(\omega_n - \omega) + \gamma_n^{(P)} + \Gamma_n^{(P)}} ,$$

In the limit of a symmetric cavity, the reflection, transmission, and absorption for each polariton branch are then given by

$$R_n(\omega) = \frac{(\omega_n - \omega)^2 + \left(\Gamma_n^{(P)}\right)^2}{(\omega_n - \omega)^2 + \left(\gamma_n^{(P)} + \Gamma_n^{(P)}\right)^2} , \tag{S.5a}$$

$$T_n(\omega) = |it(\omega)|^2 = \frac{\left(\gamma_n^{(P)}\right)^2}{(\omega_n - \omega)^2 + \left(\gamma_n^{(P)} + \Gamma_n^{(P)}\right)^2} , \tag{S.5b}$$

$$A_n(\omega) = 1 - R(\omega) - T(\omega) = \frac{2\gamma_n^{(P)}\Gamma_n^{(P)}}{(\omega_n - \omega)^2 + \left(\gamma_n^{(P)} + \Gamma_n^{(P)}\right)^2} , \tag{S.5c}$$

resulting in the polaritonic Elliott formula (Eq. 4 of the main text). These equations also apply to the case of a bare Fabry-Pérot cavity by setting $\Gamma_n^{(P)} = 0$ and replacing $\gamma_n^{(P)}$ with the bare cavity decay rate κ. This allows us to fit and extract the cavity photon linewidth from the S-matrix simulation, as shown by the black dashed curve in the inset of Fig.S.2. Furthermore, in the excitonic limit, $\Gamma^{(P)} = \Gamma$ and $\gamma^{(P)} = \gamma$, with γ being the radiative decay rate of the exciton, equation S.5c gives the well-known excitonic Elliott formula[17]. In this case, excitons can couple directly to the ports, rather than indirectly via cavity photons. The Elliott formula reveals that the maximum absorption of 0.5 at resonance is reached when the critical coupling condition, $\Gamma_n^{(P)} = \gamma_n^{(P)}$, is met[11–13,15].

Comparing the absorption of the single perovskite layer between the S-matrix method and the Elliott formula in Fig. S.4, we find excellent agreement for all states. The minor

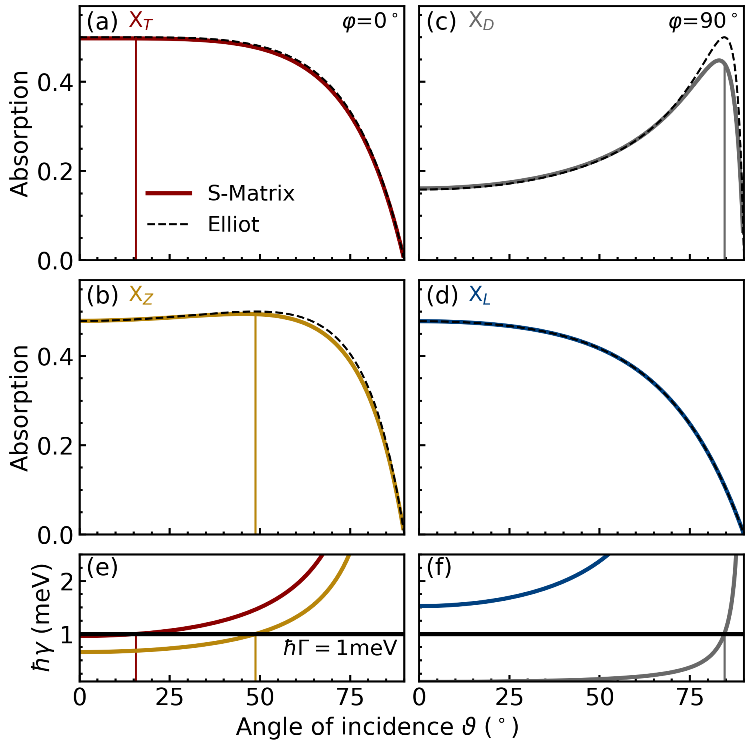

Figure S.4: (a)-(d) Absorption of a $(PEA)_2PbI_4$ perovskite layer as a function of the angle of incidence, ϑ, resonant with the four exciton fine structure states X_T, X_Z, X_D and X_L calculated using the S-matrix method (colored solid lines) and the Elliott formula (black dashed lines). (e)-(f) Radiative decay of the respective states (colored lines) and material-based loss (black horizontal line) as a function of ϑ for the states shown in (a)-(d), respectively. The colored vertical lines illustrate, where the excitonic decay rate equals the respective photonic decay, i.e., where the critical coupling condition is met resulting in a maximum absorption.

deviation reflects a small spectral overlap between the X_T and X_Z excitons, which is not taken into account in the Elliott formula. Additionally, we find that the critical coupling condition fully describes the absorption behavior, as illustrated by the vertical colored lines in Fig. S.4.

S.2 Polariton landscape

Without a magnetic field, only the two circularly polarized bright states X_T and X_L can couple to the TE-polarized light, as shown in Figs. S.1(a)-(b). Therefore, only two azimuth-angle-independent polariton branches are visible in absorption, cf. Fig. S.5. In other words,

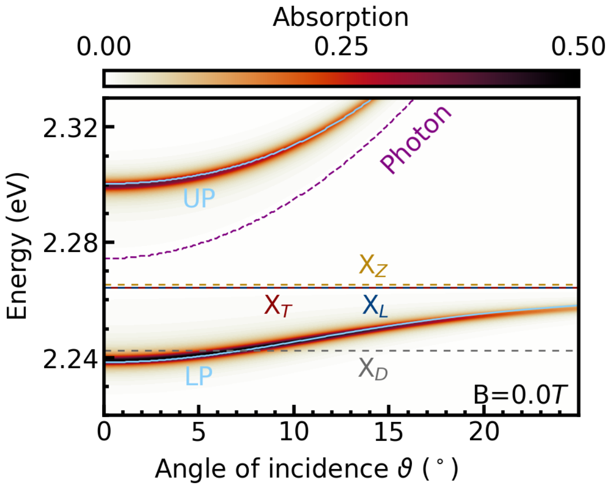

Figure S.5: Absorption of a 2D $(PEA)_2PbI_4$ perovskite layer integrated within a Fabry-Pérot microcavity as a function of the photon energy and angle of incidence at $B = 0\,\mathrm{T}$. The dashed horizontal lines denote the energy of the different excitonic states. As only the degenerate bright states X_T and X_L can couple to the light (due to the selection rules shown in Fig. S.1), only two polariton branches, LP and UP, are visible with a fully dark middle polariton branch lying on top of the two degenerate states. As there is no breaking of in-plane symmetry, the absorption spectrum is independent of the azimuth angle.

the polariton dispersion is isotropic in the absence of a magnetic field. These two branches are equivalent to the polaritons visible in Fig. 3(a) of the main text, showing a single large Rabi splitting centered on X_T. This is because even at $B = 50\,\mathrm{T}$, the two transversally polarized states X_T and X_Z are still almost degenerate in energy and have together the same in-plane oscillator strength as X_T at $B = 0\,\mathrm{T}$ due to the conservation of oscillator strength, cf. Fig. S.1(b).

The dependence of the polariton landscape on the magnetic field strength is shown in Fig. S.6. Similar to Fig. 3 in the main text, it reveals a single branch (LP) around the energy of the dark state at $\varphi = 0°$, two at $\varphi = 90°$ (LP' and MP') and a superposition of the two edge cases at $\varphi = 45°$ with approximately halved absorption. The MP' branch can only be resolved starting at about $10\,\mathrm{T}$ because, below this field strength, the dark state lacks sufficient oscillator strength to enter the strong coupling regime, i.e., $g < (\hbar\gamma + \hbar\Gamma)/2$. The energies of LP' and MP' polaritons are used to calculate the Rabi splitting around the dark state as a function of B, shown in Fig. 4(a) of the main text.

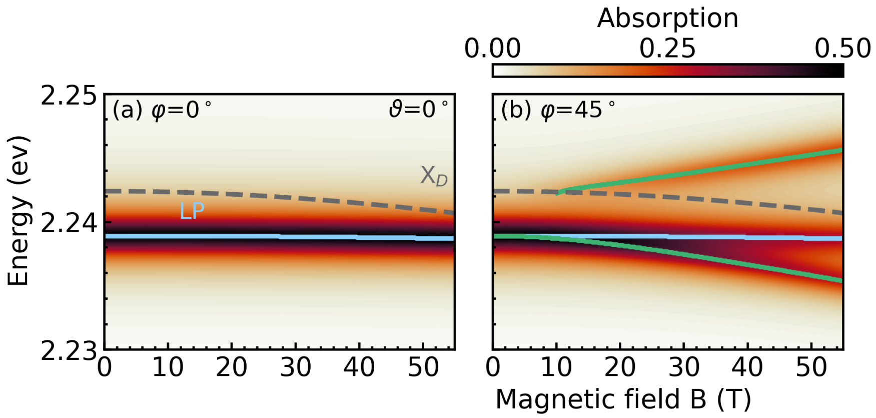

Figure S.6: (a)-(c) Absorption of a 2D $(PEA)_2PbI_4$ perovskite layer integrated within a Fabry-Pérot microcavity as a function of the photon energy and magnetic field strength B, for $\vartheta = 0°$, and for three different azimuth angles $\varphi = 0°$, 45°, and 90°. The dashed line denotes the energy of the dark state X_D. As the latter can not couple to light at $\varphi = 0°$, according to the selection rules, only a single polariton branch (LP) stemming from the energetically higher bright states can be observed in this energy range. In contrast, for $\varphi = 90°$, the dark state can couple to the cavity mode, and therefore two branches appear (LP' and MP').

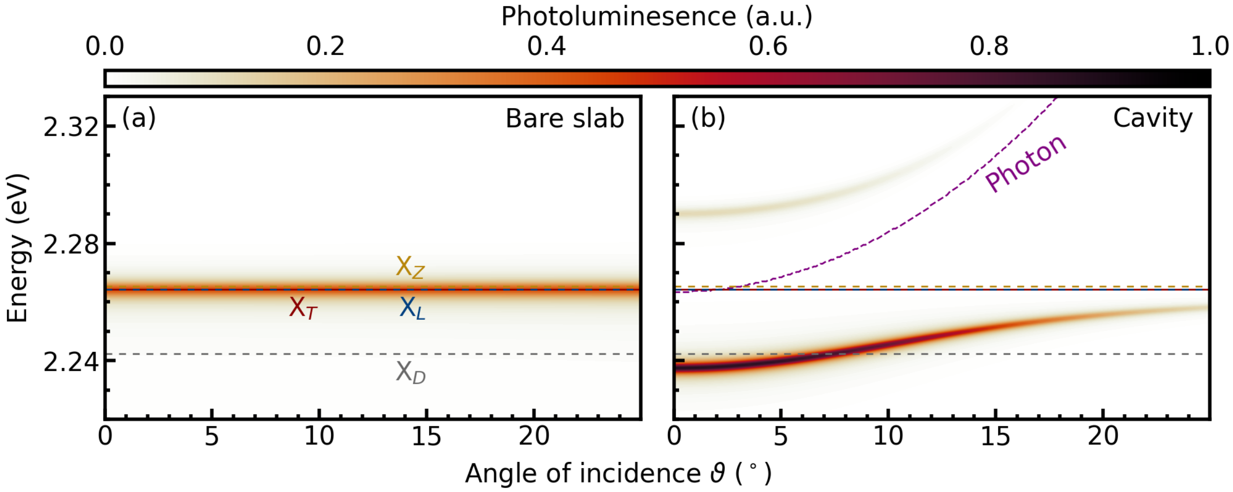

Figure S.7: (a) Emission of a bare 2D $(PEA)_2PbI_4$ perovskite layer as a function of the photon energy and angle of incidence $\vartheta = 0°$ at $B = 0\,\mathrm{T}$. The dashed line denotes the energy of the exciton states. (b) same as (a) but for the perovskite layer intgrated within a Fabry-Pérot microcavity. The purple dashed line denotes the cavity photon dispersion. Now the energetically lowest state is not the dark state X_D, but the lower polariton branch, resulting in a much higher photoluminescence. The detuning was chosen such that the cavity mode aligns with the bright exciton states at $\vartheta = 0°$.

S.3 Brightening of a dark material

While the dark exciton state X_D remains optically inactive at $B = 0\,\mathrm{T}$, it is noteworthy that the material can still be brightened through strong light–matter coupling. As shown in Fig. S6, the coupling between the two degenerate bright exciton states X_T and X_L with the cavity mode forms an upper and lower polariton branch. For certain detuning ranges, the lower polariton can be positioned energetically below the dark exciton state, eliminating the need for magnetic-field-induced splitting to achieve brightening[18].

This mechanism is reflected in the photoluminescence (PL) emission results shown in Fig. S.7(a), where the bare layer exhibits low PL due to the dark exciton state being optically inactive. However, once the system enters the strong coupling regime, as illustrated in Fig. S.7(b), the formation of polariton states shifts the system to a regime where strong emission is favorable from the lower polariton due to an increased occupation. The PL intensity calculations are based on the Kubo-Martin-Schwinger relation[19], which links the emission to the absorption spectrum multiplied by a Boltzmann distribution[20]. This is valid for thermal equilibrium, high temperatures, and no bottleneck effects.

S.4 Hopfield coefficients

The Hopfield coefficients for the polariton branches shown in Fig. 3 of the main text are obtained by fitting the Hopfield/CMT model to the reflection spectrum and shown Fig. S.8. Both lower branches, LP and LP', have the highest contribution from their respective excitons in the small-incident-angle limit, while in the high-angle limit, they become almost fully photonic. The two upper branches, UP and UP' show the opposite behavior: they possess the highest photonic contribution at small angles and are almost fully excitonic at high angles[15]. For $\varphi = 0°$, the middle polariton branch, MP, has a negligible photonic component and therefore appears flat and almost dark, cf. Fig. 3(a) of the main text. In contrast, the MP' branch for $\varphi = 90°$ exhibits a photonic component and thus has a sizable absorption

(Fig. 3(c) of the main text). These Hopfield coefficients at normal incidence, $\vartheta = 0°$, are used to obtain the material-based and radiative polariton linewidths, $\hbar\Gamma_n^{(P)}$ and $\hbar\gamma^{(P)}$ respectively, which help explain the absorption shown in Fig. 4 of the main text.

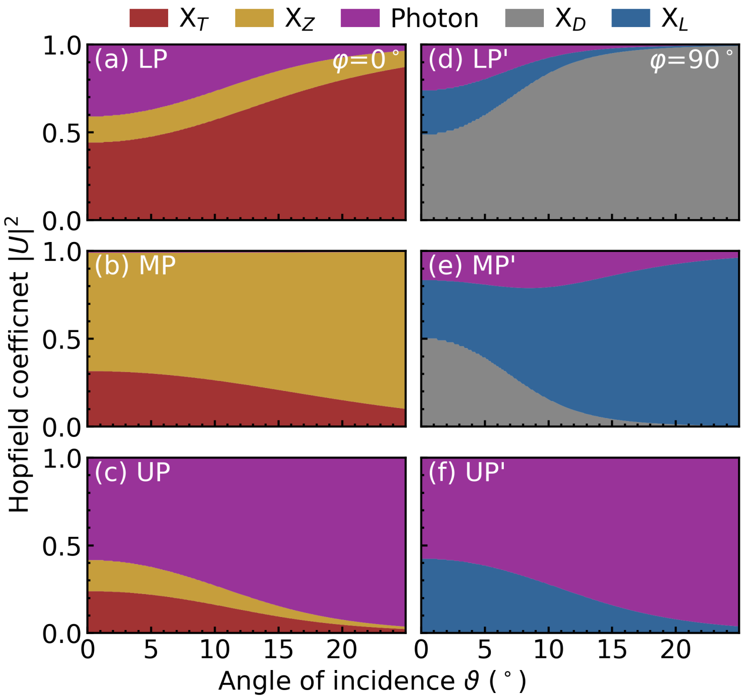

Figure S.8: Hopfield coefficients as a function of angle of incidence, ϑ, for the polariton branches shown in Figs. 3(a) and (c) of the main text. They describe the excitonic and photonic composition of these branches.

References

(1) Brem, S.; Selig, M.; Berghaeuser, G.; Malic, E. Exciton relaxation cascade in two-dimensional transition metal dichalcogenides. *Scientific reports* **2018**, *8*, 1–8.

(2) Ziegler, J. D.; Zipfel, J.; Meisinger, B.; Menahem, M.; Zhu, X.; Taniguchi, T.; Watanabe, K.; Yaffe, O.; Egger, D. A.; Chernikov, A. Fast and anomalous exciton diffusion in two-dimensional hybrid perovskites. *Nano Letters* **2020**, *20*, 6674–6681.

(3) Feldstein, D.; Perea-Causin, R.; Wang, S.; Dyksik, M.; Watanabe, K.; Taniguchi, T.; Plochocka, P.; Malic, E. Microscopic picture of electron–phonon interaction in two-

dimensional halide perovskites. *The Journal of Physical Chemistry Letters* **2020**, *11*, 9975–9982.

(4) Hong, X.; Ishihara, T.; Nurmikko, A. Dielectric confinement effect on excitons in PbI 4-based layered semiconductors. *Physical Review B* **1992**, *45*, 6961.

(5) Thompson, J. J.; Dyksik, M.; Peksa, P.; Posmyk, K.; Joki, A.; Perea-Causin, R.; Erhart, P.; Baranowski, M.; Loi, M. A.; Plochocka, P.; others Phonon-Bottleneck Enhanced Exciton Emission in 2D Perovskites. *Advanced Energy Materials* **2024**, *14*, 2304343.

(6) Dyksik, M.; Duim, H.; Maude, D. K.; Baranowski, M.; Loi, M. A.; Plochocka, P. Brightening of dark excitons in 2D perovskites. *Science advances* **2021**, *7*, eabk0904.

(7) Rumpf, R. C. Improved formulation of scattering matrices for semi-analytical methods that is consistent with convention. *Progress In Electromagnetics Research B* **2011**, *35*, 241–261.

(8) Rodríguez-de Marcos, L. V.; Larruquert, J. I.; Méndez, J. A.; Aznárez, J. A. Self-consistent optical constants of SiO 2 and Ta 2 O 5 films. *Optical Materials Express* **2016**, *6*, 3622–3637.

(9) Fieramosca, A.; De Marco, L.; Passoni, M.; Polimeno, L.; Rizzo, A.; Rosa, B. L.; Cruciani, G.; Dominici, L.; De Giorgi, M.; Gigli, G.; others Tunable out-of-plane excitons in 2D single-crystal perovskites. *Acs Photonics* **2018**, *5*, 4179–4185.

(10) Fan, S.; Suh, W.; Joannopoulos, J. D. Temporal coupled-mode theory for the Fano resonance in optical resonators. *JOSA A* **2003**, *20*, 569–572.

(11) Ferreira, B.; Rosati, R.; Fitzgerald, J. M.; Malic, E. Signatures of dark excitons in exciton–polariton optics of transition metal dichalcogenides. *2D Materials* **2022**, *10*, 015012.

(12) König, J. K.; Fitzgerald, J. M.; Hagel, J.; Erkensten, D.; Malic, E. Interlayer exciton polaritons in homobilayers of transition metal dichalcogenides. *2D Materials* **2023**, *10*, 025019.

(13) Ferreira, B.; Shan, H.; Rosati, R.; Fitzgerald, J. M.; Lackner, L.; Han, B.; Esmann, M.; Hays, P.; Leibeling, G.; Watanabe, K.; Taniguchi, T.; Eilenberger, F.; Tongay, S.; Schneider, C.; Malic, E. Revealing Dark Exciton Signatures in Polariton Spectra of 2D Materials. *ACS Photonics* **2024**, *11*, 2215–2220.

(14) Hopfield, J. Theory of the contribution of excitons to the complex dielectric constant of crystals. *Physical Review* **1958**, *112*, 1555.

(15) Fitzgerald, J. M.; Thompson, J. J.; Malic, E. Twist angle tuning of moiré exciton polaritons in van der Waals heterostructures. *Nano Letters* **2022**, *22*, 4468–4474.

(16) Zhao, Z.; Guo, C.; Fan, S. Connection of temporal coupled-mode-theory formalisms for a resonant optical system and its time-reversal conjugate. *Physical Review A* **2019**, *99*, 033839.

(17) Kira, M.; Koch, S. W. Many-body correlations and excitonic effects in semiconductor spectroscopy. *Progress in quantum electronics* **2006**, *30*, 155–296.

(18) Shan, H.; Iorsh, I.; Han, B.; Rupprecht, C.; Knopf, H.; Eilenberger, F.; Esmann, M.; Yumigeta, K.; Watanabe, K.; Taniguchi, T.; others Brightening of a dark monolayer semiconductor via strong light-matter coupling in a cavity. *Nature communications* **2022**, *13*, 3001.

(19) Chatterjee, S.; Ell, C.; Mosor, S.; Khitrova, G.; Gibbs, H.; Hoyer, W.; Kira, M.; Koch, S.; Prineas, J.; Stolz, H. Excitonic photoluminescence in semiconductor quantum wells: Plasma versus excitons. *Physical review letters* **2004**, *92*, 067402.

(20) Shan, H.; Fitzgerald, J. M.; Rosati, R.; Leibeling, G.; Watanabe, K.; Taniguchi, T.; Tongay, S. A.; Eilenberger, F.; Esmann, M.; Höfling, S.; others Tuning relaxation and nonlinear upconversion of valley-exciton-polaritons in a monolayer semiconductor. *arXiv preprint arXiv:2505.00385* **2025**,
